# General duality and magnet-free passive phononic Chern insulators

**DOI:** 10.1038/s41467-023-36420-4

**Published:** 2023-02-17

**Authors:** Qicheng Zhang, Li He, Eugene J. Mele, Bo Zhen, A. T. Charlie Johnson

**Affiliations:** 1 Department of Physics and Astronomy, University of Pennsylvania, Philadelphia, PA 19104 USA; 2 Department of Materials Science and Engineering, University of Pennsylvania, Philadelphia, PA 19104 USA

**Keywords:** Condensed-matter physics, Acoustics, Topological insulators, NEMS

## Abstract

Integrated phononics plays an important role in both fundamental physics and technology. Despite great efforts, it remains a challenge to break time-reversal symmetry to achieve topological phases and non-reciprocal devices. Piezomagnetic materials offer an intriguing opportunity as they break time-reversal symmetry intrinsically, without the need for an external magnetic field or an active driving field. Moreover, they are antiferromagnetic, and possibly compatible with superconducting components. Here, we develop a theoretical framework that combines linear elasticity with Maxwell’s equations via piezoelectricity and/or piezomagnetism beyond the commonly adopted quasi-static approximation. Our theory predicts and numerically demonstrates phononic Chern insulators based on piezomagnetism. We further show that the topological phase and chiral edge states in this system can be controlled by the charge doping. Our results exploit a general duality relation between piezoelectric and piezomagnetic systems, which can potentially be generalized to other composite metamaterial systems.

## Abstract (web summary)

Understanding and controlling symmetry in nature is of paramount importance. In this work, the authors reveal an unexpected effect of the general duality relation between piezoelectricity and piezomagnetism on their symmetries, enabling novel phononic Chern insulators.

## Introduction

Electromagnetic (EM) duality is a generic symmetry of Maxwell’s equations in free space or any combination of materials^1^ with a fixed ratio between permittivity *ϵ* and permeability *μ*. The EM duality demonstrates that, with appropriate normalization, Maxwell’s equations are invariant under the transformation of: **E**→**H**, **H**→−**E**. Here **E** is the electric field, and **H** is the auxiliary field. When the ratio of *ϵ/μ* is not a constant in space, this duality is broken. Nevertheless, we note that a “general duality”^2, 3^ can still be preserved in matter, as defined by the replacements:

1 $$\left(\epsilon,\,\mu {{{{{\rm{;}}}}}}\,{{{{{\bf{E}}}}}},\,{{{{{\bf{H}}}}}}\right)\to \left(\mu,\,\epsilon {{{{{\rm{;}}}}}}\,{{{{{\bf{H}}}}}},\,-{{{{{\bf{E}}}}}}\right)$$

In fact, this general duality, which maps one system described by (*ϵ*, *μ*) to a different system with (*μ*, *ϵ*), is always preserved, as long as no charges are present; namely, ∇·**D** = ∇·**B** = 0. Beyond EM systems, general duality can be further extended to composite systems, where EM fields are coupled to other degrees of freedom. One example is the general duality between piezoelectric (PZE) systems, where the strain field **ε** is coupled to the electric field (***ε*** = *S****σ*** *+* *d*_*e*_**E**, **σ** is the stress field)^4, 5^, and piezomagnetic (PZM) systems, where strain is coupled to the auxiliary field (***ε*** *=* *S****σ*** *+* *d*_*m*_**H**)^6, 7^. Specifically, this general duality is captured by the transformation:

2 $$\left(\epsilon,\,\mu,\, {d}_{e},\,{d}_{m}{{{{{\rm{;}}}}}}\,{{{{{\boldsymbol{\sigma }}}}}},\,{{{{{\bf{E}}}}}},\,{{{{{\bf{H}}}}}}\right)\to \left(\mu,\,\epsilon,\,{d}_{m},\,-{d}_{e}{{{{{\rm{;}}}}}}\,{{{{{\boldsymbol{\sigma }}}}}},\,{{{{{\bf{H}}}}}},\,{{{{{\rm{-}}}}}}{{{{{\bf{E}}}}}}\right)$$

The transformed solution of (**σ**, **H**, −**E**) is a solution to the new system, defined by (*μ, ϵ, d*_*m*_*, −d*_*e*_) if (**σ**, **E**, **H**) is a solution to the original system described by (*ϵ, μ, d*_*e*_*, d*_*m*_). Furthermore, the two solutions share the same frequency.

While this general duality transformation may seem straightforward from the mathematical point of view, it leads to interesting physical consequences in terms of symmetry and topology. For example, breaking time-reversal symmetry ${{{{{\mathcal{T}}}}}}$ is a necessary (but not sufficient) condition^8^ for non-trivial Chern numbers, allowing for the Chern insulator phases. But in some ${{{{{\mathcal{T}}}}}}$-broken cases, the general duality strictly forbids Chern insulator phase. Specifically, a PZM system^9, 10^ necessarily breaks ${{{{{\mathcal{T}}}}}}$, but preserves inversion symmetry, ${{{{{\mathcal{P}}}}}}$, allowing the Chern insulator phase; meanwhile, a PZE system^5^ preserves ${{{{{\mathcal{T}}}}}}$ but breaks ${{{{{\mathcal{P}}}}}}$, forbidding the Chern insulator phase. The two systems, having very different symmetries, support fundamentally different topological phases, yet the duality connection between their fields requires them to share the same topological invariants, such as Berry curvatures and Chern numbers. As a result, we find all phononic bands have zero Chern number, even if ${{{{{\mathcal{T}}}}}}$ is broken in PZM systems.

Here we apply this general duality to develop insights into PZE and PZM systems as well as phononic Chern insulators. Since PZE systems preserve ${{{{{\mathcal{T}}}}}}$, we find that an effective time-reversal symmetry $\widetilde{{{{{{\mathcal{T}}}}}}}$ emerges in PZM systems via the general duality, and this symmetry protects degeneracies and prevents the opening of topological bandgaps. We further show that doping charges can break both the general duality and $\widetilde{{{{{{\mathcal{T}}}}}}}$, enabling the design of phononic Chern insulators that support backscattering-immune chiral edge states. In contrast to standard methods to break reciprocity, our approach does not require an external magnetic field^11–17^ or driving field^18–24^, making it potentially useful in low-power or low-temperature radio frequency applications.

## Results

### Master equation and the general duality

We start by deriving the master equation that couples linear elasticity to electromagnetic waves via the PZE and PZM effects. Our theoretical framework extends beyond the commonly adopted quasi-static approximation^4, 7^. It reads:

3 $${{{{{\mathcal{H}}}}}}{{{{{\boldsymbol{\psi }}}}}}=i{\partial }_{t}{{{{{\mathcal{B}}}}}}{{{{{\boldsymbol{\psi }}}}}}$$

$${{{{{\mathcal{H}}}}}} 	=\left[\begin{array}{cccc} & i{M}_{\partial }^{T} & & \\ i{M}_{\partial } & & iq{I}_{3} & \\ & -{iq}{I}_{3} & & i\nabla \times \\ & & -i\nabla \times & \end{array}\right]{{{{{\mathcal{,}}}}}}\,{{{{{\mathcal{B}}}}}}{{{{{\mathcal{=}}}}}}\left[\begin{array}{cccc}S & & {d}_{e} & {d}_{m}\\ & \rho & & \\ {d}_{e}^{{{\dagger}} } & & \epsilon & \\ {d}_{m}^{{{\dagger}} } & & & \mu \end{array}\right],\\ {M}_{\partial }	=\left[\begin{array}{cccccc}{\partial }_{x} & & & & {\partial }_{z} & {\partial }_{y}\\ & {\partial }_{y} & & {\partial }_{z} & & {\partial }_{x}\\ & & {\partial }_{z} & {\partial }_{y} & {\partial }_{x} & \end{array}\right]$$

The master equation can be viewed as an eigenvalue problem, with ${{{{{\boldsymbol{\psi }}}}}}$ = [**σ**, **v**, **E**, **H**]^*T*^ being the eigenstate, where **u** is the mechanical displacement and **v**, the velocity, **v** = ∂_t_**u**; *S*, the mechanical compliance matrix; *ρ*, the mass density; *I*_*3*_, rank-3 identity matrix; and *q*, the doping charge density. A detailed derivation of Eq. 3 is included in Section 1 of the Supplementary Information.

This set of equations can be intuitively understood as follows: the first row represents the constitutive relationship between ***σ***, **v**, **E**, and **H**, taking into account PZE and PZM effects. See Supplementary Information Section 2 for a simplified model of the PZM effect. The second row is the elastic equation of motion to linear order. The third and fourth rows are the modified Maxwell’s equations, taking into account doping charges and the PZE and PZM responses. We note that our model allows coupling to doping charges and is, thus, different from the master equation for common dielectric materials: the electric field applies forces on charged dopants, leading to stress and induced strain in the material. The reversed process is also included in the model, where stress causes doped charges to move and produce an EM field. Neither of these processes exist in standard dielectric materials where the charge separation is too small to have effect on acoustic phonons. The examples covered in the main text include *d*_*m*_ real with *d*_*e*_ = 0, and *d*_*e*_ real with *d*_*m*_ = 0. Systems where both *d*_*m*_ and *d*_*e*_ are non-zero are of the Tellegen type and have been considered by others^25, 26^. The case of complex valued *d*_*m*_ is discussed in the Section 3 of the Supplementary Information.

The time-reversal operator ${{{{{\mathcal{T}}}}}}$ associated with the master equation is defined as

4 $${{{{{\mathcal{T}}}}}}={{{{{\rm{diag}}}}}}\left([I,-I,\, I,-I]\right){{{{{\mathcal{K}}}}}}$$

where ${{{{{\mathcal{K}}}}}}$ is complex conjugation. Each *I* in the diagonal block represents an identity matrix with the dimension of ***σ***, **v**, **E**, **H**, respectively. The alternating pattern between +*I* and −*I* arises from the fact that, under ${{{{{\mathcal{T}}}}}}$, stress ***σ*** and electric field **E** remain unchanged, but velocity **v** and auxiliary field **H** reverse direction. It is straightforward to show that the metric matrix ${{{{{\mathcal{B}}}}}}$ is ${{{{{\mathcal{T}}}}}}$-invariant, $\left[{{{{{\mathcal{B}}}}}}{{{{{\mathcal{,}}}}}}{{{{{\mathcal{T}}}}}}\right]=0$, if and only if the PZM effect is absent, i.e., *d*_*m*_ = 0. It is therefore clear that PZM breaks ${{{{{\mathcal{T}}}}}}$ ^9, 10^.

Next, we describe the general duality between PZE and PZM in the context of the master equation. In the absence of doping charge, *q*, Eq. 3 can be rewritten, using a unitary transformation

$${U}_{d}=\left[\begin{array}{cccc}1 & & & \\ & 1 & & \\ & & & 1\\ & & -1 & \end{array}\right]$$

as: ${{{{{\mathcal{H}}}}}}$**ψ**′ = i∂_t_${{{{{{\mathcal{B}}}}}}}^{{\prime} }$ **ψ**′. Here, **ψ**′ = U_d_**ψ**, and ${{{{{{\mathcal{B}}}}}}}^{{\prime} }={U}_{d}{{{{{\mathcal{B}}}}}}{U}_{d}^{-1}$. Note that the original system, described by ${{{{{\mathcal{B}}}}}}$*(ϵ, μ, d*_*e*_*, d*_*m*_), is now mapped onto a new system, described by ${{{{{\mathcal{B}}}}}}$^*'*^*(μ, ϵ, d*_*m*_*, −d*_*e*_*)*; meanwhile the ${{{{{\mathcal{H}}}}}}$ matrix is invariant under the transformation *U*_*d*_.

Since *U*_*d*_ is unitary, the eigenvalue spectra for the two systems are identical. For example, for a pure PZM system (*d*_e_ = 0,*d*_m_ ≠ 0) the dual system becomes ${U}_{d}{{{{{\mathcal{B}}}}}}\left(\epsilon,\,\mu,\,0,\,{d}_{m}\right){U}_{d}^{-1}{{{{{\mathcal{=}}}}}}{{{{{\mathcal{B}}}}}}\left(\mu,\,\epsilon,\,{d}_{m},\,0\right)$, which is a pure PZE system, but sharing the same eigenvalue spectra as the original system, up to linear order. Applying the unitary transformation to the eigenstates, the original solution of **ψ** = (**σ**, **v**, **E**, **H**) is mapped to **ψ**′ = (**σ**, **v**, **H**, −**E**). We note that the two solutions share the same topological invariant of Berry curvature, so the two systems share the same Chern number, while ${{{{{\mathcal{B}}}}}}$ is ${{{{{\mathcal{T}}}}}}$-broken and allowed non-zero Chern number but ${{{{{\mathcal{B}}}}}}$ is ${{{{{\mathcal{T}}}}}}$-preserved and requests zero Chern number. See Section 4 in the Supplementary Information for a detailed derivation.

Aside from eigenvalues and eigenstates, the general duality also connects the symmetries satisfied by each system. For example, the time reversal symmetry ${{{{{\mathcal{T}}}}}}$ preserved in a purely PZE system is transformed to an equivalent symmetry of

5 $$\widetilde{{{{{{\mathcal{T}}}}}}}={U}_{d}^{-1}{{{{{\mathcal{T}}}}}}{U}_{d}={{{{{\rm{diag}}}}}}\left([{{{{{\rm{I}}}}}},-{{{{{\rm{I}}}}}},-{{{{{\rm{I}}}}}},\, {{{{{\rm{I}}}}}}]\right){{{{{\mathcal{K}}}}}}$$

in the dual purely PZM system. Next, we show that both ${{{{{\mathcal{T}}}}}}$ and $\widetilde{{{{{{\mathcal{T}}}}}}}$ need to be broken to achieve a Chern insulator phase in a PZM system, which is a direct consequence of the general duality.

### Magnet-free passive phononic Chern insulators

To study topological phases in a PZM system, we develop a numerical method to solve the master equation, assuming *d*_*e*_ = 0 in all cases below. We start by lifting Dirac degeneracies in the band structure of a periodically patterned PZM material. In a purely elastic (***σ***, **v**) or EM (**E**, **H**) wave system, it is known that Dirac degeneracies occur at the corners of the Brillouin zone of a honeycomb lattice and are protected by the symmetry ${{{{{\mathcal{P}}}}}}{{{{{\mathcal{T}}}}}}$^8^. For the PZM system, ${{{{{\mathcal{P}}}}}}$ is preserved while ${{{{{\mathcal{T}}}}}}$ is broken, so one might assume that the Dirac degeneracy will be lifted, leaving an energy gap in the spectrum. However, we show below that this intuition is incorrect.

**Fig. 1:**
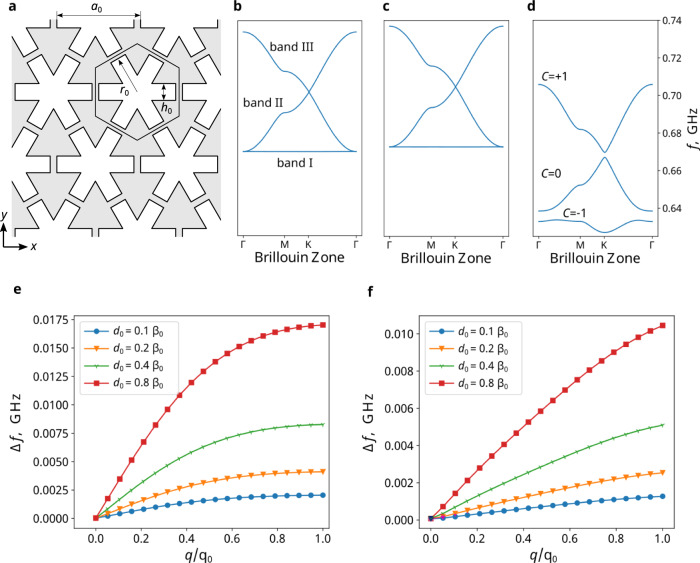
Time-reversal symmetry breaking in charge-doped PZM phononic crystals. **a** Schematic of the phononic crystal design. Within each hexagonal unit cell, a snowflake-shaped white region is etched away. The geometrical parameters (a_0_ = 2.3 μm, r_0_ = 1 μm, h_0_ = 0.5 μm) are kept the same for panels. The band structure is varied when the bulk material changes from (**b**) a pure elastic material to (**c**) a charge neutral PZM material and to (**d**) a PZM material with a uniform doping charge density *q*. A Chern insulator is achieved in **d**, where bandgaps are opened. The bandgap size variation with charge doping density *q* at different PZM coupling strength *d*_0_ (**e**) between band I/II at the Γ point, and (**f**) between band II/III at the *K* point. *d*_0_ is the magnitude of nonzero elements of *d*_*m*_, normalized by β_0_ = 5.9 nm/A, and q_0_ = 232.5 C/mm^3^.

To confirm this, we numerically solve the eigenvalue problem associated with Eq. 3 for a phononic crystal (Fig. 1a), which has *D*_*6h*_ symmetry in the *xy*-plane. A periodic boundary condition is applied in the *z*-direction (out of plane) with *k*_*z*_ = 0. We start with the simplest case of a pure elastic structure: in the absence of PZM effect (*d*_*m*_ = 0) or doping charges (*q* = 0), both ${{{{{\mathcal{H}}}}}}$ and ${{{{{\mathcal{B}}}}}}$ are block diagonal, separating (**σ**, **v**) from (**E**, **H**). This means that the elastic wave and EM wave are decoupled, as expected. We focus on the low-frequency phonon branches. Protected by symmetries, degeneracies are found within the Brillouin zone, both between bands I and II at the Γ point and between bands II and III at the K and K’ points forming two Dirac points (Fig. 1b). These results are consistent with the symmetry analysis of Dirac points provided above.

The introduction of the PZM effect and charged dopants couples the elastic and EM waves and modifies the system’s band structure. For simplicity, we choose *d*_*m*_ terms which block diagonalize ${{{{{\mathcal{H}}}}}}$ and ${{{{{\mathcal{B}}}}}}$, while focusing on the waves involving (transverse) phonon components vibrating asymmetrically with respect to the *xy-*plane, and the transverse-magnetic EM components with non-vanishing *E*_*z*_, *H*_*x*_, and *H*_*y*_ (Supplementary Information Section 5). Since preserving or breaking ${{{{{\mathcal{T}}}}}}$ does not depend on the details of *d*_*m*_, this simplification does not affect the discussions. Interestingly, PZM alone, though it breaks ${{{{{\mathcal{T}}}}}}$, does not lift the Dirac degeneracy (Fig. 1c). However, when both PZM and charge doping (*q*) are present, Dirac degeneracies are lifted and bandgaps are created, both between bands I and II and between bands II and III (Fig. 1d). At low charge doping levels, both bandgaps increase linearly with *q* (Fig. 1e), emphasizing its important role in achieving non-trivial topological phases. We note that only the existence of doping charges, not a net charge, is required to gap out Dirac points, as shown in a later example with zero net charge in Fig. 4.

The reason why both PZM and charge doping are required to open the energy gap is due to the general duality. For a ${{{{{\mathcal{T}}}}}}$-broken PZM system without charge doping (*q* = 0), a dual PZE system can be created, which shares the same energy spectrum, Berry curvature, and Chern number as the original PZM system. Since the dual PZE system is ${{{{{\mathcal{T}}}}}}$-symmetric, all bands are required to have zero Chern number, which must also be true for the original PZM system that is ${{{{{\mathcal{T}}}}}}$-broken. This can also be understood using effective symmetries: time reversal symmetry ${{{{{\mathcal{T}}}}}}$, preserved in the dual PZE system, is translated into a new symmetry of $\widetilde{{{{{{\mathcal{T}}}}}}}={U}_{d}^{-1}{{{{{\mathcal{T}}}}}}{U}_{d}$, which is preserved in the original PZM system. ${{{{{\mathcal{P}}}}}}\widetilde{{{{{{\mathcal{T}}}}}}}$ protects the Dirac degeneracy for the PZM system, even though ${{{\mathcal{P}}}}{{{\mathcal{T}}}}$ is broken. The only way to lift the degeneracy at the Dirac points is to include charges (*d*_*m*_ ≠ 0, *q* ≠ 0), which breaks the general duality and thus $\widetilde{{{{{{\mathcal{T}}}}}}}$, as shown in Fig. 1d–f. The newly opened gaps feature non-zero Chern numbers and support unidirectional chiral edge states (CESs) for phonons. The calculated Chern numbers (Supplementary Information Section 4) are shown for bands I, II, and III in Fig. 1d. Accordingly, both bandgaps are topological and support CESs as shown next.

**Fig. 2:**
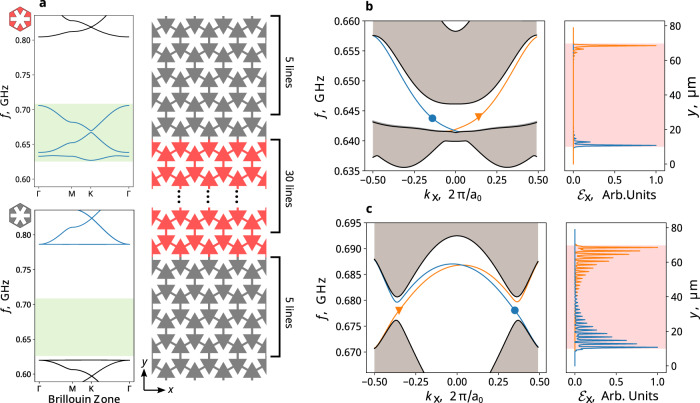
Magnet-free passive phononic Chern insulator and chiral edge states. **a** The supercell setup for edge state calculation. Left: the band structures of the red and black phononic crystal unit cells. The red unit cell consists of PZM materials with positive doping charges, and the black unit cell consists of dielectric materials with Young’s modulus of 36.5 GPa, featuring a trivial gap within the frequency of interest shaded in green. Right: schematic drawing of the supercell, which consists of 40 unit cells in the *y-*direction and is periodic along x-direction. The dispersion relations (left panels) and mode profiles (right) of the marked states are shown for the chiral edge states within the lower (**b**) and the higher frequency gap (**c**). The edge states are localized at the bottom (blue) or top (orange) interface as shown in their energy density *E*_*x*_.

A defining feature of a Chern insulator is the existence of CESs at its interface with a trivial insulator, determined by the bulk-edge correspondence^27^. Here, we calculate the CES dispersion using a super-cell setup as shown in Fig. 2a. Specifically, a Chern insulator region (red), using the same geometry as in Fig. 1d is surrounded by a trivial phononic insulator on the top and bottom (black). Through the design of the black unit cell, both topological gaps lie within the trivial energy gap. Periodic boundary conditions are applied in both *x* and *y* directions. The supercell modes are numerically computed as shown in Fig. 2b, c, which consist of both bulk modes (area shaded in gray) and edge modes (orange and blue lines). Bulk-edge correspondence is confirmed for both topological gaps: for the lower-frequency gap with C = −1 (Fig. 2b), two edge states are observed, one (orange) located at the top edge traveling to the left, and the other one (blue) located at the bottom edge traveling to the right. For the higher frequency gap, also with C = −1 (Fig. 2c), a pair of non-trivial edge states with the same chirality are also observed.

**Fig. 3:**
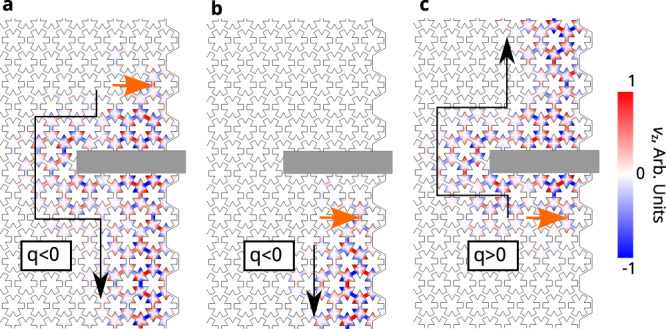
Unidirectional and backscattering-immune phonon transport through CESs. **a**, **b** CESs excited by a point source at the interface between a phononic Chern insulator and air above and below the large scatterer (gray region), when the bulk has a negative doping charge density (*q* < 0). **c** The CES propagates along the opposite direction when the bulk doping charge density is positive (*q* > 0). The blue-red color scheme represents the out-of-plane velocity *v*_*z*_.

Next, we show that the CESs are immune to backscattering and that their chirality can be controlled by the sign of doping charges in the bulk. As shown in Fig. 3, a point source at frequency 0.645 GHz is placed near the right interface between a Chern insulator (*q* < 0) with the same design as in Fig. 2b and air. This frequency is in the lower energy gap with C = −1 in Fig. 2b. In all calculations (Fig. 3a–c), the phononic crystal is terminated by absorbing boundary conditions along the top, bottom, and left edges to eliminate back-reflection. The right edge is terminated by air, which acts like a trivial bandgap for the phonon modes of interest. The gray region is a defect that consists of a harder, purely elastic material. A CES is excited by a point force source and propagates in the clockwise direction determined by the Chern number of the bulk (Fig. 3a). It travels around the defect region and keeps propagating with perfect transmission, as it is protected by the bulk topology. The comparison between Fig. 3a, b shows the clear signature of non-reciprocal transport through CESs: phonon waves can only propagate downward, but not upward. We further show that the chirality of the edge states is controlled by the sign of doping charges. As shown in Fig. 3c, the edge state switches direction and propagates upward instead, when the doping charge is changed from negative to positive. This further confirms the importance of doping charges in achieving Chern insulators: *q* = 0 is the phase transition point between two Chern insulators with opposite Chern numbers. Accordingly, the energy gap has to close at *q* = 0, which is consistent with the results presented in Fig. 1.

The fact that the sign of *q* controls the edge state chirality can be understood from the perspective of $\widetilde{{{{{{\mathcal{T}}}}}}}$. Although *q* is ${{{{{\mathcal{T}}}}}}$-even, it acts as an effective magnetic field since it breaks the effective time-reversal symmetry $\widetilde{{{{{{\mathcal{T}}}}}}}$, and in this way induces the Chern insulator phase. Interestingly, in the PZE system, *q* behaves like an effective electric field and flips valley degree of freedom (Supplementary Information Section 6) similar to what happens in bilayer graphene^28^. The dual functions of *q*, acting as an effective magnetic field in PZM system and as effective electric field in PZE system, come from the fact that it breaks the general duality.

## Discussion

**Fig. 4:**
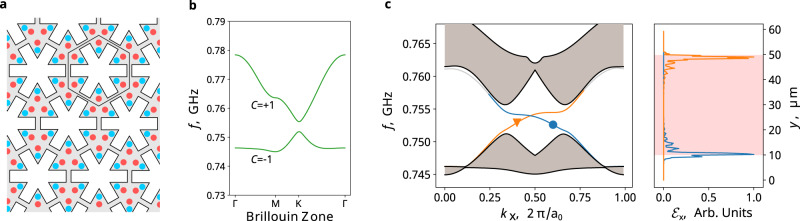
Phononic Chern insulators in a net-charge-neutral PZM structure. **a** Schematic drawing of a PZM phononic crystal with both positive (red) and negative charges (blue), giving rise to a net zero charge per unit cell. **b** Band structure of the PZM phononic crystal featuring two topological bands with C = ±1. **c** Simulation results showing CESs between the net-charge-neutral phononic Chern insulator and harder elastic material like Fig. 2a.

We note that only the existence of doping charges, not a net charge, is required to open topological gaps. One such example is shown in Fig. 4, where both positive (red) and negative (blue) charged dopants are introduced in a phononic unit cell in a symmetric way, giving rise to a net-charge-neutral PZM phononic crystal. In this charge neutral system, topological gaps can still be opened (Fig. 4b, green bands), supporting unidirectional CESs (Fig. 4c). As expected in such system, the size of the topological gap depends on both the magnitude of the charges and their separations: the positive-negative-charge separation should not be much smaller than the lattice constant of the phononic crystal to be effective, otherwise, the Coulomb force cannot effectively distinguish between the charges or modify the elastic wave. This observation is consistent with the fact that our examples focus on the phonon branches with low frequencies and long wavelengths. Details of the charge dopant distribution and band calculations are provided in Supplementary Information Section 7.

Finally, we discuss the feasibility of experimentally realizing these proposed structures and phononic Chern insulators. The compositive wave can be excited by interdigital transducers like in Ref. ^17^. The key parameters are doping charge density *q* and piezomagnetic response *d*_*m*_. Our estimates are based on results in Fig. 1e, f. The charge *q* is normalized by the value q_0_ = 232.5 C/mm^3^, while non-zero elements of *d*_*m*_, with typical magnitude *d*_0_, are normalized to β_0_ = 5.9nm/A (see Methods for additional information regarding how these normalization values are chosen). Doping charges can be achieved by ion implantation, where the effective density can be as high as 5 × 10^17^ ions/cm^2^ ^29^. For a 100 nm-thick film, the normalized charge q/q_0_ can be as high as 0.03; while for a 10 nm thin film, q/q_0_ can reach 0.3. PZM materials typically have d_0_/β_0_ on the order of 0.01^30^. Assuming the gap size varies linearly with q/q_0_ and d_0_/β_0_, we estimate a bandgap of 0.1 MHz with existing PZM materials at q/q_0_ = 0.3. Further development of PZM materials to larger d_0_/β_0_ will lead to larger bandgap. Another method to achieve even larger topological gaps is to use magnetostrictive materials that are closely related to piezomagnetism, which can achieve a bandgap larger than 10 MHz, but require a finite, though small, external magnetic field or a residual magnetization^31^. See Section 8 of Supplementary Information for more details. In addition to the material parameters, the optimization of mode profiles and PZM coupling terms at different degeneracy points can enlarge the bandgaps and strengthen the localization of edge states.

In summary, we have theoretically proposed and numerically demonstrated integrated non-reciprocal phononic devices that do not require external driving fields or magnetic fields. This may make them uniquely suitable for radio-frequency signal processing and use in quantum technologies incorporating superconducting devices. Our theoretical framework enhances the scientific understanding of PZE and PZM effects, opens possibilities of vibrational engineering through charge doping, and can guide the implementation of other topological phases in integrated phononic crystals^32–35^. The concepts of general dualities and derived hidden symmetries also promise additional physical insights when extended to other systems with general dualities^2, 36^.

## Methods

### Simulation of band structures and spatial mode profiles

The simulation is done using finite element method by writing Eq.3 as weak form in COMSOL Multiphysics 5.4. The geometric parameters of the system are a_0_ = 2.3 μm, r_0_ = 1 µm, and h_0_ = 0.5 µm. The following isotropic mechanical material parameters of Terfenol-D are used: Youngs modulus 26.5 GPa, Poisson ratio 0.3, mass density ρ_0_ = 9100 kg/m^3^. The d_m_ term (PZM effect) is normalized by ${{{{{{\rm{\beta }}}}}}}_{0}=\sqrt{{{{{{{\rm{\mu }}}}}}}_{0}/{{{{{{\rm{c}}}}}}}_{11}}=5.9\times {10}^{-9}\,{{{{{\rm{m}}}}}}/{{{{{\rm{A}}}}}}$, where the μ_0_ is the vacuum permittivity and c_11_ = 35.67 GPa is the *xxxx* element of the elastic matrix. The doping charge density is normalized by ${{{{{{\rm{q}}}}}}}_{0}=\frac{2\pi }{{{{{{{\rm{a}}}}}}}_{0}}\,\sqrt{\frac{{{{{{{\rm{\rho }}}}}}}_{0}}{{{{{{{\rm{\mu }}}}}}}_{0}}}\approx 232.5{C}/{{{{{\rm{m}}}}}}{{{{{{\rm{m}}}}}}}^{3}$.

## Supplementary information

Supplementary Information

## Data Availability

All data supporting the findings of this study are available within the article and/or the SI Appendix. The raw data are available from the corresponding author upon reasonable request.

## Code availability

The codes are available from the corresponding author upon reasonable request.
