# Supplementary material for: General duality and magnet-free passive phononic Chern insulators
Source: Nat Commun. 2023 Feb 17;14:916. doi: 10.1038/s41467-023-36420-4 (PMC9938148; doi:10.1038/s41467-023-36420-4)
Supplement: Supplementary file 1 — Supplementary Information [file 41467_2023_36420_MOESM1_ESM.pdf]

Supplementary Information

General duality and magnet-free passive phononic Chern insulators

Qicheng Zhang[1], Li He[1], Eugene Mele[1], Bo Zhen[1,*], A. T. Charlie Johnson[1,2,*]

[1]Department of Physics and Astronomy, University of Pennsylvania, Philadelphia 19104, USA
[2]Department of Materials Science and Engineering, University of Pennsylvania, Philadelphia, PA

19104, USA

*Emails: bozhen@sas.upenn.edu; cjohnson@physics.upenn.edu

Section 1: Derivation of the master equation

First, we would like to show that pure elastic problem can be formulated as Eq. 3, where $\boldsymbol{\psi}_{el} = [\boldsymbol{\sigma},\ \mathbf{v}]^T$ and

$$\mathcal{H}_{\text{el}} = \begin{bmatrix} 0 & iM_\partial^T \\ iM_\partial & 0 \end{bmatrix}, \mathcal{B}_{\text{el}} = \begin{bmatrix} S & 0 \\ 0 & \rho \end{bmatrix}.$$

The first row of the equation is $M_\partial^T \mathbf{v} = M_\partial^T \partial_t \mathbf{u} = \partial_t M_\partial^T \mathbf{u} = \partial_t\ \boldsymbol{\varepsilon} = \partial_t\ (S\boldsymbol{\sigma})$. Here $\boldsymbol{\varepsilon} = S\boldsymbol{\sigma}$ is the constitutive equation. The vector $\boldsymbol{\sigma}$ and $\boldsymbol{\varepsilon}$ used are tensor σ, ε written in Voigt notation,

$$\boldsymbol{\sigma} = \left[\sigma_{xx}, \sigma_{yy}, \sigma_{zz}, \sigma_{yz}, \sigma_{xz}, \sigma_{xy}\right]^T,\ \ \boldsymbol{\varepsilon} = \left[\varepsilon_{xx}, \varepsilon_{yy}, \varepsilon_{zz}, 2\varepsilon_{yz}, 2\varepsilon_{xz}, 2\varepsilon_{xy}\right]^T = M_\partial^T \left[u_x, u_y, u_z\right]^T.$$

The tensor form $\varepsilon_{ij} = \frac{1}{2}\left(u_{i,j} + u_{j,i}\right)$. The subscript comma represents derivation $u_{i,j} = \partial_j u_i$, the subscript index runs on $\{x, y, z\}$. The Voigt notation further gives $[\nabla \cdot \boldsymbol{\sigma}]_i = \partial_j \sigma_{ij} = [M_\partial \boldsymbol{\sigma}]_i$. So, we can write the second row of the matrix equation as $M_\partial \boldsymbol{\sigma} = \nabla \cdot \boldsymbol{\sigma} = \partial_t \rho \mathbf{v}$ using the elastic equation of motion $\nabla \cdot \boldsymbol{\sigma} = \partial_t \rho \mathbf{v}$ to the linear order.

Maxwell's equations also be written in the form[1] of Eq. 3 with $\boldsymbol{\psi}_{mw} = [\mathbf{E}, \mathbf{H}]^T$ and

$$\mathcal{H}_{\text{mw}} = \begin{bmatrix} 0 & i\nabla\times \\ -i\nabla\times & 0 \end{bmatrix}, \mathcal{B}_{\text{mw}} = \begin{bmatrix} \epsilon & 0 \\ 0 & \mu \end{bmatrix}.$$

Combining $\boldsymbol{\psi} = [\boldsymbol{\psi}_{el}; \boldsymbol{\psi}_{mw}]$ with the PZE and PZM constitutive relationship

$$\boldsymbol{\varepsilon} = S\boldsymbol{\sigma} + d_e\mathbf{E} + d_m\mathbf{H}$$

$$\mathbf{D} = d_e^T\boldsymbol{\sigma} + \epsilon\mathbf{E}$$

$$\mathbf{B} = d_m^T\ \boldsymbol{\sigma} + \mu\mathbf{H}$$

The $\mathcal{B}$ matrix becomes:

$$\mathcal{B} = \begin{bmatrix} S & 0 & d_e & d_m \\ 0 & \rho & 0 & 0 \\ d_e^T & 0 & \epsilon & 0 \\ d_m^T & 0 & 0 & \mu \end{bmatrix}.$$

Here d_e and d_m are real. The $\mathcal{H}$ part needs to incorporate the effect of doping charges. The elastic equation of motion $\nabla \cdot \boldsymbol{\sigma} = \partial_t \rho \mathbf{v}$ comes from the balance of momentum, and the introduction of doping charges resulted in a body force (Coulomb force) which modifies the equation of motion to $\nabla \cdot \boldsymbol{\sigma} + q\mathbf{E} = \partial_t \rho \mathbf{v}$. On the other hand, the movement of doped charges creates a current which appears in Maxwell's equations as $\nabla \times \mathbf{H} = \partial_t \mathbf{D} + q\mathbf{v}$, here $\mathbf{D}$ is the electric displacement field. These effects modify the overall $\mathcal{H}$ to

$$\mathcal{H} = \begin{bmatrix} 0 & iM_\partial^T & 0 & 0 \\ iM_\partial & 0 & iqI_3 & 0 \\ 0 & -iqI_3 & 0 & i\nabla\times \\ 0 & 0 & -i\nabla\times & 0 \end{bmatrix}.$$

Next, we will show that by allowing complex amplitude for vector $\boldsymbol{\psi}$, $\mathcal{B} = \mathcal{B}^\dagger$ for a lossless system. We only show the PZM effect while the PZE effect can be derived similarly.

The energy balance for the EM waves is $\frac{d\mathcal{E}_{EM}}{dt} = -\nabla \cdot \mathbf{S}_P - q\mathbf{v} \cdot \mathbf{E}$. Here $\mathcal{E}_{EM}$ is the EM wave energy and $\mathbf{S}_P$ is the Poynting vector. On the other hand, the transport equation of mechanical energy gives $\frac{d\mathcal{E}_{el}}{dt} = \nabla \cdot (\sigma\mathbf{v}) + q\mathbf{E} \cdot \mathbf{v}$, where the $\mathcal{E}_{el}$ is the mechanical energy. In the coupled lossless system, the time average of the total energy in a period $\frac{1}{T}\int_0^T dt\, \left(\frac{d\mathcal{E}_{EM}}{dt} + \frac{d\mathcal{E}_{el}}{dt}\right) = \frac{1}{T}\int_0^T dt\, \left(-\nabla \cdot \mathbf{S}_P + \nabla \cdot (\sigma\mathbf{v})\right) = 0$. By substituting in components in $\boldsymbol{\psi} = Re\left(\boldsymbol{\psi}_m e^{i\omega t}\right) = \frac{1}{2}\left(\boldsymbol{\psi}_m e^{i\omega t} + \boldsymbol{\psi}_m^* e^{-i\omega t}\right)$, using the first order time derivation equations listed in Eq. 3, we get:

$$\mathbf{p}_m \cdot \mathbf{v}_m^* - \mathbf{p}_m^* \cdot \mathbf{v}_m + \sigma_m^*\varepsilon_m - \sigma_m\varepsilon_m^* + \mathbf{B}_m \cdot \mathbf{H}_m^* - \mathbf{B}_m^* \cdot \mathbf{H}_m + \mathbf{D}_m \cdot \mathbf{E}_m^* - \mathbf{D}_m^* \cdot \mathbf{E}_m = 0.$$

Combining with the complex constitutive relationship:

$$\varepsilon_m = S\sigma_m + \zeta\mathbf{H}_m, \qquad \mathbf{p}_m = \rho\mathbf{v}_m, \qquad \mathbf{B}_m = \xi\,\sigma_m + \mu\mathbf{H}_m, \qquad \mathbf{D}_m = \epsilon\mathbf{E}_m,$$

We have $S = S^\dagger, \rho = \rho^\dagger, \epsilon = \epsilon^\dagger, \mu = \mu^\dagger, \zeta = \xi^\dagger$.

Therefore, the Eq. 3 can be written as:

$$\mathcal{H} = \begin{bmatrix} 0 & iM_\partial^T & 0 & 0 \\ iM_\partial & 0 & iqI_3 & 0 \\ 0 & -iqI_3 & 0 & i\nabla\times \\ 0 & 0 & -i\nabla\times & 0 \end{bmatrix}, \mathcal{B} = \begin{bmatrix} S & 0 & 0 & d_m \\ 0 & \rho & 0 & 0 \\ 0 & 0 & \epsilon & 0 \\ d_m^\dagger & 0 & 0 & \mu \end{bmatrix}.$$

Section 2: A simplified model of PZM effect

PZM materials intrinsically break $\mathcal{T}$.[2–4] This can be understood from that the PZM tensor is $\mathcal{T}$-odd, since it links the $\mathcal{T}$-even strain tensor (rank two) and the $\mathcal{T}$-odd magnetic field vector. To give a better physical understanding of PZM effect, the schematic drawing of a simplified model is shown in Fig. S1. Magnets with alternating directions are fixed onto a mechanical lattice, in which grey bars with equal lengths are joined freely. Giving a strong alignment interaction which fixes the directions of the magnets to the lattice, a linear relationship between the magnetic moment and the strain is established. The sum of exchange energy and Zeeman energy in this system is

$\frac{3}{2}J\ \mathbf{M}_1 \cdot \mathbf{M}_2 - \mu_0 \mathbf{H} \cdot (\mathbf{M}_1 + \mathbf{M}_2) = 3JM_0^2\left(\varepsilon^2 - \frac{1}{2}\right) - 2\mu_0 M_0 H_x \varepsilon$. The symbol meanings are: J, exchange coupling term constant; $\mathbf{M_1}$ ($\mathbf{M_2}$), the magnetic moment which points up (down) in the strain-free lattice; M_0, absolute value of the magnetic moment of a single magnet; $\varepsilon = \cos\theta$, the mechanical strain and θ is the angle defining the mechanical lattice configuration as show in Fig. 1c; μ_0, vacuum permeability; H_x, external magnetic field along x direction. The constitutive equation of stress is therefore a derivative against strain, $\sigma = 6JM_0^2\varepsilon - 2\mu_0 M_0 H_x$. We see that in additional to the elastic term, there is a PZM coupling term $2\mu_0 M_0$ changing sign under $\mathcal{T}$, which flips the staggered magnetization M_0.

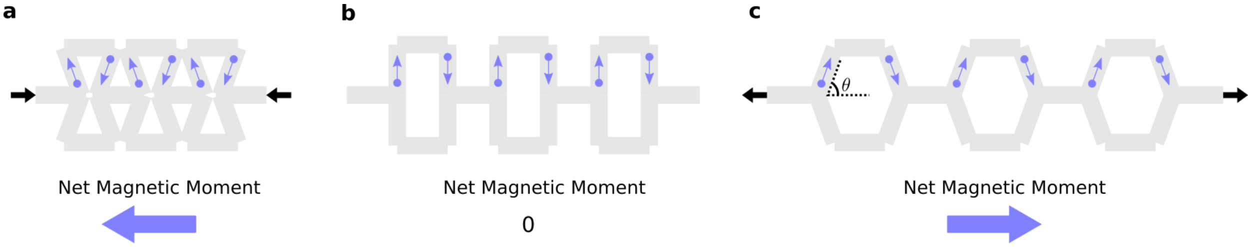

Figure S1| A simplified model of PZM effect. Equal length bars (grey) are joined freely at heads and tails. Magnets (small purple arrows) are fixed on some bars with their directions aligned with the grey bars. The net magnetic moment changes with stress, in (**a**) compressed, **(b)** free, and (**c**) stretched states. The strain ε is determined by the angle θ illustrated in (**c**).

Section 3: Complex valued d_m in the presence of general duality

At $q = 0$, $\mathcal{H}$ is block diagonal, this makes it possible to put a phase difference between $(\boldsymbol{\sigma}, \mathbf{v})$ and $(\mathbf{E}, \mathbf{H})$. Therefore, if d_m is pure imaginary, it can be mapped back to a real d_m case. When d_m is complex in generally (note this is a matrix) and cannot be reduced to a real d_m, it usually means there are some dynamics coupled. For example, when net magnetization is induced, the precession gives both real and imaginary part in the susceptibility, making d_m effectively complex[5]. Generally, it can be numerically verified that if a precession is presented, the topological bandgap can be opened. However, this case is physically different from the case with real d_m. When d_m is real, the net magnetic moment is 0 and the $\mathcal{T}$-broken order parameter is the "staggered magnetization"[6]. The staggered magnetization determines d_m (also see the simplified model in Section 2). When d_m is complex, the order parameter is the non-zero magnetic moment. The operator U_d does not have a very significant consequence in the latter case, where a complex d_m

is mapped to a complex d_e (PZE coupling when there is a rotation of polarization). Both complex d_m and d_e open a topological bandgap because a generic angular momentum is introduced in both cases and is a traditional way to break $\mathcal{T}$.

Section 4: Transformation of Chern number and symmetries

From Eq. 3, we can easily find $\mathcal{H}^\dagger = \mathcal{H}, \mathcal{B}^\dagger = \mathcal{B}$ and operator $i\partial_t$ commute with $\mathcal{B}$ since we do not apply any dynamic active driving field. So, Eq. 3 is Hermitian. By defining the Hilbert space inner product $\langle\psi_i|\psi_j\rangle = \int dV\ \psi_i^\dagger \mathcal{B}\psi_j$ with matrix $\mathcal{B}$ as the metric, the Chern number of this system can be calculated as generalized Hermitian eigenvalue problem[7]. The Berry connection can be directly adapted as $\boldsymbol{\mathcal{A}} = -i\,\langle\phi|\nabla_{\mathbf{k}}|\phi\rangle$. Here ϕ is the spatial periodical part of Bloch wave $\boldsymbol{\psi}$. The Berry curvature is then $F = \nabla_{\mathbf{k}} \times \boldsymbol{\mathcal{A}}$ and the Chern number of a separate band is the integral of Berry curvature over the first Brillouin zone $C = \frac{1}{2\pi}\int_{BZ} F \cdot ds$.

We note that U_d (defined in the main text) is a unitary transformation which preserves the Hilbert space inner product $\langle\psi_i{}'|\psi_j{}'\rangle = \int dV\ \psi_i^\dagger U_d^\dagger\ U_d \mathcal{B} U_d^{-1}\ U_d \psi_j = \int dV\ \psi_i^\dagger \mathcal{B}\psi_j = \langle\psi_i|\psi_j\rangle$, and that U_d is independent of $\mathbf{k}$. Therefore, U_d also preserves the Berry connection $\boldsymbol{\mathcal{A}}$ as $\boldsymbol{\mathcal{A}}' = -i\,\langle\phi' \mid \nabla_{\mathbf{k}} \mid \phi'\rangle = -i\,\langle\phi|U_d^\dagger \nabla_{\mathbf{k}} U_d|\phi\rangle = -i\,\langle\phi|\nabla_{\mathbf{k}}|\phi\rangle = \boldsymbol{\mathcal{A}}$. Consequently, Berry curvature and Chern number are invariant under U_d.

Operator U_d maps symmetries from one system to its dual system.

All the possible systems are characterized by space $\mathrm{L}(\mathcal{B}) = \{\mathcal{B}\}$ which is a set of Hermitian matrices $\mathcal{B}$. We limit our discussion to real values of $L(\mathcal{B})$. We can also define subspaces $L_{pzm} = \{\mathcal{B}|d_e = 0, d_m \neq 0\}$ and $L_{pze} = \{\mathcal{B}|d_e \neq 0, d_m = 0\}$, corresponding to PZM and PZE spaces. Using the unitary operator U_d defined in the main text, we have $U_d^{-1} L_{pze} U_d = L_{pzm}$. This makes it possible to "copy" a symmetry from L_{pze} to L_{pzm} at $q = 0$.

If a symmetry of L_{pze} is labeled g_e,

$$\forall\, \mathcal{B}_e \in L_{pze},\ \ g_e \mathcal{B}_e g_e^{-1} = \mathcal{B}_e.$$

Then $U_d^{-1} g_e U_d$ is a symmetry of L_{pzm}. This is because

$$\forall \mathcal{B}_m \in L_{pzm},\ \ \mathcal{B}_e' = U_d \mathcal{B}_m U_d^{-1} \in L_{pze},$$

$$(U_d^{-1} g_e U_d)\mathcal{B}_m(U_d^{-1} g_e^{-1} U_d) = U_d^{-1} g_e \mathcal{B}_e' g_e^{-1} U_d$$

$$= U_d^{-1} \mathcal{B}_e' U_d = \mathcal{B}_m$$

By using U_d, L_{pzm} can "copy" the symmetry $\mathcal{T}$ from L_{pze} so:

$$\tilde{\mathcal{T}} = U_d^{-1}\mathcal{T}U_d$$

Reversely, L_{pze} can "copy" symmetry $\mathcal{P}$ from L_{pzm}:

$$\tilde{\mathcal{P}} = U_d\mathcal{P}U_d^{-1}$$

Section 5: The block diagonalization of the asymmetric and symmetric modes

The $\boldsymbol{\psi}$ vector is composed of 15 elements, including 6 stress tensor elements, 3 velocity vector elements, 3 electric field vector elements and 3 magnetic field vector elements. However, under certain circumstances, Eq. 3 can be block diagonalized which greatly simplifies the calculation. First, $\partial_z = 0$ reduces the rank of $\mathcal{H}$ and remove σ_3 from $\boldsymbol{\psi}$. If we re-arrange the elements in $\boldsymbol{\psi}$ as $[\sigma_4, \sigma_5, v_z;\ E_z, H_x, H_y;\ \sigma_1, \sigma_2, \sigma_6, v_x, v_y;\ E_x, E_y, H_z]$, we can block diagonalize $\mathcal{H}$. Here the elements are rearranged as [asymmetric vibration mode; transverse electric field mode; symmetric vibration mode; transverse magnetic field mode]. If we assume d_m terms are all real, with isotropic mechanical, EM properties, $\mathcal{B}$ becomes:

$$\begin{bmatrix}
S_{44} & & & & [d_m]_{14} & [d_m]_{24} & & & & & & & & [d_m]_{34} \\
& S_{55} & & & [d_m]_{15} & [d_m]_{25} & & & & & & & & [d_m]_{35} \\
& & \rho & & & & & & & & & & & \\
& & & \epsilon_3 & & & & & & & & & & \\
[d_m]_{14} & [d_m]_{15} & & & \mu_1 & & [d_m]_{11} & [d_m]_{12} & [d_m]_{16} & & & & & \\
[d_m]_{24} & [d_m]_{25} & & & & \mu_2 & [d_m]_{21} & [d_m]_{22} & [d_m]_{26} & & & & & \\
& & & & [d_m]_{11} & [d_m]_{21} & S_{11} & S_{12} & & & & & & [d_m]_{31} \\
& & & & [d_m]_{12} & [d_m]_{22} & S_{21} & S_{22} & & & & & & [d_m]_{32} \\
& & & & [d_m]_{16} & [d_m]_{26} & & & S_{66} & & & & & [d_m]_{36} \\
& & & & & & & & & \rho & & & & \\
& & & & & & & & & & \rho & & & \\
& & & & & & & & & & & \epsilon_1 & & \\
& & & & & & & & & & & & \epsilon_2 & \\
[d_m]_{34} & [d_m]_{35} & & & & & [d_m]_{31} & [d_m]_{32} & [d_m]_{36} & & & & & \mu_3
\end{bmatrix}$$

In the simulations, we only use $[d_m]_{14}$, $[d_m]_{15}$, $[d_m]_{24}$, $[d_m]_{25}$. Therefore, $\mathcal{B}$ is also block diagonal. For simplicity, we only use asymmetric vibration components (transverse wave) and TM components for all simulations ($[\sigma_4, \sigma_5, v_z;\ E_z, H_x, H_y]$). We keep C_6 symmetry (here we use[8] $(d_m)_{14} = -(d_m)_{25} = (d_m)_{24} = (d_m)_{15} = d_0$). The Eq. 3 becomes:

$$\begin{bmatrix} & & i\partial_y & & & \\ & & i\partial_x & & & \\ i\partial_y & i\partial_x & & & & \\ & & & & -i\partial_y & i\partial_x \\ & & & -i\partial_y & & \\ & & & i\partial_x & & \end{bmatrix}\begin{bmatrix}\sigma_4\\ \sigma_5\\ v_z\\ E_z\\ H_x\\ Hy\end{bmatrix} = i\partial_t\begin{bmatrix} S_{44} & & & & d_0 & d_0 \\ & S_{55} & & & d_0 & -d_0 \\ & & \rho & & & \\ & & & \epsilon_3 & & \\ d_0 & d_0 & & & \mu_1 & \\ d_0 & -d_0 & & & & \mu_2 \end{bmatrix}\begin{bmatrix}\sigma_4\\ \sigma_5\\ v_z\\ E_z\\ H_x\\ Hy\end{bmatrix}$$

Section 6: The effect of the general duality in the PZE system

As a direct result from the general duality, PZE system, which is a dual system of the PZM system as demonstrated in the main text, is also affected by the general duality. More specifically, in a PZE system, where $\mathcal{P}$ is broken but $\mathcal{T}$ is preserved, the general duality creates $\tilde{\mathcal{P}} = U_d \mathcal{P} U_d^{-1}$ by mapping the $\mathcal{P}$ symmetry from the PZM system. To show the effect, we numerically solve the modes of phononic crystals with similar setup to that in the main text. In this case we focus on phonon modes vibrating symmetrically with respect to the *xy*-plane, which are coupled to the transverse-electric EM modes with non-vanishing field components of E_x, E_y and H_z. By doing so, we can use PZE parameters which preserve C_3 symmetry and couples to q. More specifically, we choose[8] $(d_e)_{11} = -(d_e)_{12} = -(d_e)_{16}/2 = (d_e)_{22} = -(d_e)_{21} = -(d_e)_{26}/2$ and other d_e terms are zero.

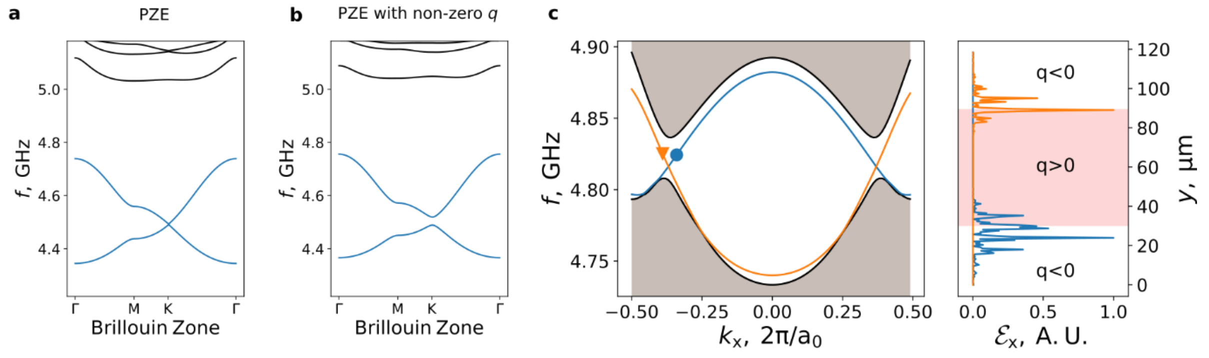

Figure S2| Effect of charge doping in PZE systems. The phononic crystal band structure with PZE effect at (**a**) $q = 0$ and (**b**) $q \neq 0$. **c**, the dispersion (left) and mode profile (right) of the VHE modes. The VHE modes localized at the bottom (top) interface are labeled blue (orange).

The 2D simulation is done with the same design as Fig. 1a in the main text. Intuitively, the Dirac point can be broken by PZE terms since $\mathcal{PT}$ symmetry is also broken in this case. However, due to the presence of $\tilde{\mathcal{P}}$ and thus $\tilde{\mathcal{P}}\mathcal{T}$, the Dirac point degeneracy lifting cannot be done by PZE terms only (Fig. S2a) and requires the presence of q (Fig. S2b). The control of the symmetry-breaking orders by q is revealed in a simulation with similar structure to Fig. 2a. In this case, we totally use 60 lines. The 30 lines in the center (red shaded in the right panel of Fig. S2c) are $q > 0$ and the other 30 lines (not shaded) are $q < 0$ while other parameters are the same. Surprisingly, the valley Hall edge (VHE) modes are observed. While VHE modes are reciprocal, they have the "valley-momentum locking" effect where the group velocity flips sign at different valleys. The appearance of VHE modes require a boundary of two regions with opposite valley configuration, usually related by inversion symmetry (or C_{2z} in 2D)[9,10] or mirror symmetry[11]. Nonetheless, charge does not flip sign in either case.

Section 7: Calculation of overall charge neutral phononic Chern insulators

In Fig. 4 of the main text, we deliberately set q to have both signs within the unit cell, where a negative charge "puddle" has the same charge density as the nearby positive one, and the charge distribution pattern keeps D_{6h} symmetry. Therefore, the sum of the doping charges over a unit cell is strictly zero. In this setup, the absolute value of q/q_0 is set to 1.0 within the puddles, much larger than what is used in the uniform charge case. The d_0 is doubled to 0.8. The super cell is set to have 20 Chern insulator unit cells and 10 trivial insulator unit cells at y direction. The material parameters of trivial insulator unit cells are the same as Fig. 1b in the main text, which puts the topological band gap at ~0.75 GHz into a trivial gap.

Section 8: Bandgap size estimation

Apart from the PZM material described in the main text. A closely- related family of materials magnetostrictive materials, operating in a modest external magnetic field of ~10 Oe, can have an effective d_0/β_0 up to as large as[12] 20. Assuming the gap size varies linearly with q/q_0 and d_0/β_0, the gap can easily become larger than 10 MHz ($d_0/\beta_0 = 1$, $q/q_0 = 0.3$ or $d_0/\beta_0 = 10$, $q/q_0 = 0.03$), which is 1.5% of the working frequency, large enough compared with the overall bandwidth

(in Fig. 1d, for example, the frequency range covered by band I, II and III areall three bands is <100 ~80 MHz). In this kind of material, loss is expected. By using Gilbert damping constant and other parameters in Ref. 12, and making both the d_m and μ terms complex according to the ferromagnetic resonance equation, we estimate the linewidth broadening at 0.68 GHz by imaginary part of the eigenvalue solution, which is ~0.2% of the central frequency. By selecting different working frequency and doing other optimizations, we expect this loss to be further reduced.

Even larger PZM effect may be achieved using metamaterials with structures like Fig. S1.

References

1. Lu, L., Joannopoulos, J. D. & Soljačić, M. Topological photonics. *Nat. Photonics* **8**, 821–829 (2014).
2. Zocher, H. & Török, C. About Space-Time Asymmetry in the Realm of Classical General and Crystal Physics. *Proc. Natl. Acad. Sci.* **39**, 681–686 (1953).
3. Dzialoshinskii, I. E. The Problem of Piezomagnetism. *Sov. J. Exp. Theor. Phys.* **6**, 621 (1958).
4. Borovik-Romanov, A. S. S. Piezomagnetism in the Antiferromagnetic Fluorides of Cobalt and Manganese. *J. Exp. Theor. Phys.* **11**, 1954 (1960).
5. Bichurin, M. I., Petrov, V. M., Kiliba, Y. V & Srinivasan, G. Magnetic and magnetoelectric susceptibilities of a ferroelectric/ferromagnetic composite at microwave frequencies. *Phys. Rev. B* **66**, 134404 (2002).
6. Jaime, M. *et al.* Piezomagnetism and magnetoelastic memory in uranium dioxide. *Nat. Commun.* **8**, 99 (2017).
7. Haldane, F. D. M. & Raghu, S. Possible Realization of Directional Optical Waveguides in Photonic Crystals with Broken Time-Reversal Symmetry. *Phys. Rev. Lett.* **100**, 13904 (2008).
8. Borovik-Romanov, A. S., Grimmer, H. & Kenzelmann, M. Piezomagnetism. *Int. Tables Crystallogr.* **D**, 133–139 (2013).
9. Martin, I., Blanter, Y. M. & Morpurgo, A. F. Topological Confinement in Bilayer Graphene. *Phys. Rev. Lett.* **100**, 036804 (2008).
10. Zhang, F., MacDonald, A. H. & Mele, E. J. Valley Chern numbers and boundary modes in

gapped bilayer graphene. *Proc. Natl. Acad. Sci.* **110**, 10546–10551 (2013).

11. Lu, J. *et al.* Observation of topological valley transport of sound in sonic crystals. *Nat. Phys.* **13**, 369 (2016).

12. Liang, X. *et al.* Soft Magnetism, Magnetostriction, and Microwave Properties of Fe-Ga-C Alloy Films. *IEEE Magn. Lett.* **10**, 1–5 (2019).
